# Evolution from genetics to phenotype: reinterpretation of NSCLC plasticity, heterogeneity, and drug resistance

**DOI:** 10.1007/s13238-016-0330-1

**Published:** 2016-10-18

**Authors:** Yingjiao Xue, Shenda Hou, Hongbin Ji, Xiangkun Han

**Affiliations:** 1 Key Laboratory of Systems Biology, Shanghai Institutes for Biological Sciences, Chinese Academy of Sciences, Shanghai, 200031 China; 2 Innovation Center for Cell Signaling Network, Shanghai Institutes for Biological Sciences, Chinese Academy of Sciences, Shanghai, 200031 China; 3 CAS Center for Excellence in Molecular Cell Science, Institute of Biochemistry and Cell Biology, Shanghai Institutes for Biological Sciences, Chinese Academy of Sciences, Shanghai, 200031 China; 4 School of Life Science and Technology, Shanghai Tech University, Shanghai, 200120 China; 5 Institute of Biochemistry and Cell Biology, Shanghai Institutes for Biological Sciences, Chinese Academy of Sciences, Shanghai, 200031 China

**Keywords:** lung cancer, plasticity, heterogeneity, drug resistance, phenotypic transition

## Abstract

Lung cancer is the leading cause of cancer-related deaths worldwide. Targeted therapy is beneficial in most cases, but the development of drug resistance stands as an obstacle to good prognosis. Multiple mechanisms were explored such as genetic alterations, activation of bypass signaling, and phenotypic transition. These intrinsic and/or extrinsic dynamic regulations facilitate tumor cell survival in meeting the demands of signaling under different stimulus. This review introduces lung cancer plasticity and heterogeneity and their correlation with drug resistance. While cancer plasticity and heterogeneity play an essential role in the development of drug resistance, the manipulation of them may bring some inspirations to cancer prognosis and treatment. That is to say, lung cancer plasticity and heterogeneity present us with not only challenges but also opportunities.

## Introduction

Lung cancer is the most common cause of cancer-related deaths in both men and women with a stable worldwide 5-year survival rate of approximately 15% despite advanced medical efforts (Key et al., 2014). Primary therapeutic strategies include surgery, chemotherapy, radiotherapy, and targeted therapy. Although these treatments can initially reduce tumor burden, relapse occurs in most cases.

Drug resistance and metastasis are the two major causes of mortality, both of which are attributed to genetic heterogeneity, genetic evolution, and tumor plasticity. Together, these three aspects produce tumor heterogeneity and allow cancer cells to survive pressures induced by dynamic changes in the surrounding microenvironment and immune system. Plasticity refers to several processes including differentiation, dedifferentiation and transdifferentiation, portraying the dramatic capacity of cells to change their fates (Shoshani and Zipori, 2015). As for cancer cells, epithelial-mesenchymal transition (EMT), and subtype transdifferentiation best embody the property of tumor plasticity. As for the arising of secondary mutations and gene amplifications against tyrosine kinase inhibitor (TKI) treatment, genetic evolution and heterogeneity theories could provide reasonable explanation. Evidences from multiple works have highlighted characteristics of cancer cell plasticity and genetic heterogeneity as a major cause of drug resistance during pre-clinical and clinical studies (Sequist et al., 2011; Shien et al., 2013). This review primarily focuses on lung cancer plasticity and heterogeneity. These facilitate cancer cell survival and bring considerable challenges to clinical treatment, yet they may also provide inspirations on cancer prognosis and potential therapies.

## Plasticity of lung cancer in epithelial to mesenchymal transition

An example of a long-known plasticity of epithelia is EMT. During cancer progression, plasticity is exemplified by morphological and phenotypical conversions such as the expression of mesenchymal markers and loss of epithelial markers (Park et al., 2015). EMT provides cancer cells with a more invasive and motile phenotype thus enhancing their invasive and metastatic potential (Thiery et al., 2009). Recent work has also demonstrated that EMT has a role in multidrug resistance (Xin et al., 2013; Sui et al., 2014).

The role of EMT in modulating cancer dissemination has been intensively addressed. Transcriptional repressors of the E-cadherin gene are activated during EMT, leading to the loss of the epithelial phenotype. The repressors can either directly repress the activity of the E-cadherin promoter or indirectly repress E-cadherin transcription (Nurwidya et al., 2012). Downstream molecules, such as Snail and ZEB, can also induce the expression of metalloproteases, which degrade the basement membrane thereby favoring invasion (Thiery et al., 2009).

EMT is also highly associated with EGFR-TKI induced drug resistance. Non-small cell lung cancer (NSCLC) cell lines sensitive to gefitinib express EMT markers such as E-cadherin, claudin-4 and claudin-7, but resistant cell lines have lost expression of these genes, while the mesenchymal marker vimentin was highly expressed in the resistant cell lines (Frederick et al., 2007). When the EMT-inducing ligand TGF-β was added to HCC4006 lung cancer cells *in vitro,* morphological changes and resistance to erlotinib were observed in two weeks. On the other hand, removal of TGF-β helped cells reverse their morphology as well as sensibility to erlotinib (Suda et al., 2011).

More specific mechanisms of EMT are still being uncovered. It has been reported that Forkhead box protein M1 (FOXM1), pyruvate dehydrogenase kinase 4 (PDK4) and Cx26 are important regulators of EMT and are associated with drug resistance (Kong et al., 2014; Sun et al., 2014; Yang et al., 2015). In a clinical study, 4 out of 9 patients with *EGFR*-mutant lung adenocarcinoma exhibited changed EMT status pre-and post-gefitinib treatments (Uramoto et al., 2010). EMT is a primary cause of metastasis, which is responsible for approximately 95% of cancer mortality. Therefore, more emphasis should be placed on understanding EMT, especially as associated with drug resistance.

## Plasticity in histological transition as a new mechanism of drug resistance

NSCLC, which accounts for 75% of all lung cancers, and small cell lung cancer (SCLC) are two subtypes of lung cancer that share distinct histological and genetic signatures. The histological heterogeneity of lung cancer makes treatment complicated. The two major subtypes of non-small cell lung cancer, lung adenocarcinoma (ADC) and squamous cell carcinoma (SCC), account for almost 80% of all subtypes of NSCLC. These subtypes can highly benefit from multiple lines of therapeutics. It is generally accepted that there are different cells-of-origin for the different types of lung cancer. For example, lung ADC is believed to be derived from type-II pneumocytes, while lung SCC descend from basal cells. High expression of neuroendocrine markers, such as calcitonin-gene related peptide (CGRP) and adhesion molecule (Ncam1) in SCLC, indicates a neuroendocrine (NE)-associated pulmonary cell-of-origin (Sutherland and Berns, 2010). To date, however, no direct evidence has identified the true cells-of-origin for all subtypes of lung cancer. Interestingly, recent clinical studies revealed that drug treatments could induce a histological transition from ADC to SCLC or SCC. These observations challenge our current knowledge on the cell-of-origin of lung cancer and further highlight the importance of cancer plasticity in lung cancer progression.

### Transformation from NSCLC to SCLC

**Figure 1:**
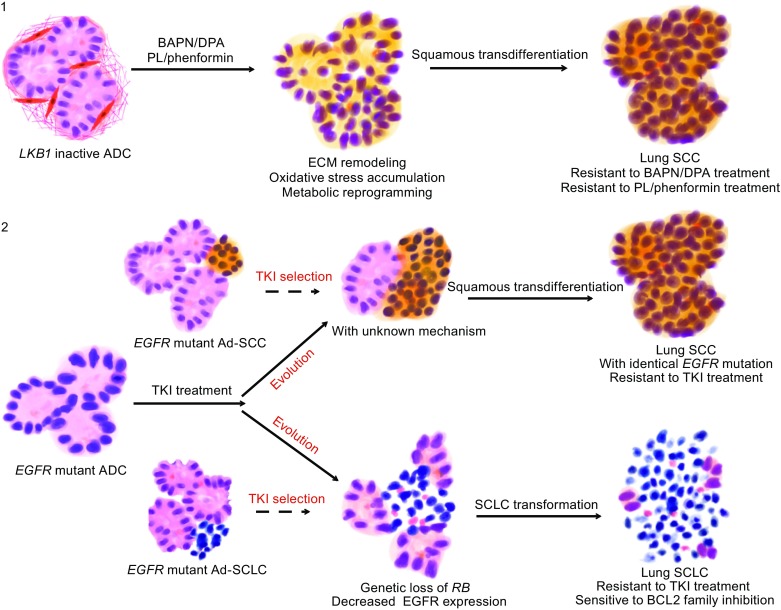
**Histological transition and drug resistance**. Lung ADC with *LKB1* deficiency treated with β-aminoproprionitrile (BAPN)  or phenformin undergoes ECM remodeling, fibroblast losing, metabolic reprograming and oxidative stress accumulation. Along with these changes the histology gradually transdifferentiates from ADC to SCC. As a consequence, the tumor eventually becomes resistant to BAPN/DPA or piperlongumine (PL)/phenformin (1). In addition, histological transition in lung *EGFR* mutant tumor also contributes to drug resistance: on one hand, ADC can transdifferentiate into SCC which is resistant to EGFR inhibitor; on the other hand, ADC can also transform into SCLC with decreased EGFR expression and resistance to EGFR inhibitor. Although the tumor still harbors identical *EGFR* mutation after histological transition, the possibility of preexistent SCC or SCLC can not be excluded (2)

Clinical observations in which primary lung ADC developed into SCLC after prolonged drug treatment (more than 2 years) have been previously reported (Morinaga et al., 2007). Engelman et al had a similar discovery. Specifically, it was reported that five patients (14%) with lung ADC were found to have a diagnosis of SCLC in EGFR TKI-resistant tumor biopsies despite maintaining the original mutation and became sensitive to classical SCLC treatment (Sequist et al., 2011). A long remission following TKI treatment and conservation of the original activating *EGFR* mutation suggests a transformation rather than a coexistence of SCLC and NSCLC. Histological transformation further supports plasticity and is one of main causes of TKI-resistance (Shien et al., 2014). In three out of the five resistant patients, genetic mechanisms of resistance were lost in the absence of the continued selective pressure of EGFR inhibitor treatment, and these cancers were sensitive to a second round of treatment with EGFR inhibitors (Sequist et al., 2011). Transformed SCLCs have features of classical SCLC including universal alterations of the RB tumor suppressor, reduced EGFR expression, and a heightened sensitivity to BCL-2 family inhibition (Niederst et al., 2015). Transformed SCLCs have *EGFR* mutations, but do not express EGFR protein, which could potentially explain why they are no longer sensitive to EGFR inhibitors. However, additional molecular mechanisms of transformation remain to be further investigated (Fig. 1).

Emma Norkowski et al found that SCLC developing with ADC, either synchronously or asynchronously, appears to be associated with *EGFR* mutations independent of TKI treatment. Two cases of NSCLC to SCLC transformation were observed after drug treatment, and two cases of phenotypic transition without TKI treatment were also observed. However, the possible coexistence of two cell types of origin could not be excluded (Norkowski et al., 2013). Although the spontaneous transformation of *EGFR* mutant ADC to SCLC may be possible, current evidence to support this are weak. Therefore, additional cases need to be analyzed to support this theory (Fig. 1).

### Transdifferentiation from lung ADC to SCC

NSCLC can be further pathologically divided into three major subtypes: ADC, SCC and large cell carcinoma (Tuveson and Jacks, 1999; Jackson et al., 2001). There is also a mixed lung adenosquamous cell carcinoma subtype (Ad-SCC) that accounts for 4–10% of NSCLC subtypes. Identical genetic mutations between the adenomatous and squamous parts of a single Ad-SCC lesion suggests that the phenotypic transition between ADC and SCC occurs according to the cancer monoclonal theory (Hofmann et al., 1994; Toyooka et al., 2006; Kang et al., 2007; Ichinokawa et al., 2011).

Stress triggered by TKI treatment has been reported as a driver of phenotype transition in clinical studies. For example, ADCs with *EGFR* mutations may transform into SCCs following TKI treatment and eventually become resistant to TKI. In another case, a mutation in *PIK3CA* has been observed in a patient with an *EGFR* mutation following erlotinib and second-line chemotherapy. Histological analysis indicated a transformation into the SCC subtype (Kuiper et al., 2015). Furthermore, transformation into SCC has been documented in two cases of *EGFR* mutated ADC with acquired resistance to gefitinib treatment (Hsieh et al., 2015). These findings emphasize the need to understand the histological changes of human lung cancer subtypes before and after drug treatment and highlight the importance of repeated biopsy during diagnosis (Fig. 1).

Genetically engineered mouse models have allowed for the extensive study of lung cancer plasticity (Sugano et al.). Han et al showed that inactivation of *Lkb1* in lung ADC conferred plasticity that promoted a progressive transformation into SCC in mice (Han et al., 2014). This process was driven by extracellular matrix (ECM) remodeling caused by downregulation of lysyl oxidase (*Lox*). Pharmacological treatment using LOX enzymatic inhibitors in *Kras* ^*G12D*^ */Lkb1* ^*L/L*^ (*KL*) mice also promoted ADC-to-SCC transition. Importantly, the SCCs remain highly proliferative following drug treatment, suggesting that this phenotypic transition could be an underlying mechanism to drug resistance in pre-clinical trials. Additionally, the authors found that reactive oxygen species (ROS) functionally modulated the ADC-to-SCC transdifferentiation in the *KL* mouse model. The squamous transition was accompanied by metabolic reprogramming upon oxidative stress accumulation during phenformin treatment in pre-clinical trials, representing a novel mechanism of drug resistance (Li et al., 2015b). It has also been demonstrated that Yes-associated protein (YAP) overexpression, through the regulation of *P63I,* inhibited ADC-to-SCC transdifferentiation, while YAP knockdown promoted this process in *Lkb1*-deficient mouse models (Gao et al., 2014). *Lkb1* deficiency causes a robust accumulation of cellular oxidative stress, which confers lung cancer cells with higher plasticity and facilitates an adaptive metabolic reprogramming of cancer cell through phenotypic transition. These works provide insight into lung cancer plasticity and cell-fate determination during tumor progression. In conclusion, histological evolution from ADC to the less stress-sensitive SCC is a strategy for tumor cells to survive and confers drug resistance (Fig. 1).

## Plasticity in chemotherapy resistance

Approximately 60% of NSCLC tumors do not harbor targetable driver mutations (Shea et al., 2016), and conventional cytotoxic chemotherapy remains the standard-of-care treatment for NSCLC patients. The most common cytotoxic agents used in treating NSCLC patients are alkylating agents, including cisplatin and carboplatin, which damage DNA by forming platinum-DNA adducts and thus disrupt DNA replication and transcription (Olaussen and Postel-Vinay, 2016). However, tumor cells adapt mechanisms to protect against cytotoxic agents, which remains an obstacle to successful treatment of NSCLC. A major mechanism of chemotherapy resistance is insufficient amount agent to reach the target DNA. For example, high expression of the detoxification protein Gluthatione S-transfer protein GSTP1 (Nakanishi et al., 1999), high expression of the plasma-membrane transporter Copper transporter 1 (CTR1) (Kim et al., 2014), and low expression of BCL-2 (Jeong et al., 2010) have been reported as mediators of resistance. Another major mechanism of resistance is failure to achieve cell death after platinum-DNA adduct formation. For instance, increased expression of ERCC1, which is a pivotal endonuclease in the nucleotide excision repair (NER) pathway (Zhou et al., 2004), and increased expression of survival signaling pathways, such as activation of the phosphatidylinositol 3 kinase (PI3K)/AKT pathway, and overexpression of HER2 (Calikusu et al., 2009) allow tumor cells to evade apoptosis. These epigenetic alterations also reflect cancer cell plasticity and enable cells to adapt to new environments caused by drug treatment. In order to enhance response and avoid resistance to chemotherapy, efforts to identify predictive biomarkers are highly needed. Moreover, selecting the appropriate chemotherapies in combination with targeted therapies or immunotherapies will be synergistic.

## Genetic evolution and heterogeneity of lung cancer in drug resistance

In addition to cancer cell plasticity, genetic alterations are a major mechanism of acquired drug resistance that has been confirmed in patients. The target gene itself can be altered by mutation or amplification, thus limiting the ability of the drug to inhibit activity of the kinase that remains active and drives aberrant signaling. Another resistance mechanism is bypass signaling, which can be activated under pressure to circumvent the inhibited kinase. Regardless of mechanism, genetic alterations lead to tumor heterogeneity and provide advantages against drug treatment.

### Drug resistance in *EGFR* mutant lung cancer

EGFR protein overexpression is frequently observed in NSCLC patients and is correlated with a poor prognosis (Hirsch et al., 2003). Hyperactivation of EGFR is closely associated with the development and progression of lung cancer. Mutation of the EGFR kinase domain accounts for the majority of EGFR activation. *EGFR* mutations occurred in 10% to 20% of NSCLC patients (Riely and Yu, 2015), so further exploration of EGFR as therapeutic target is highly valuable. Two predominant classes of EGFR inhibitors have been developed including monoclonal antibodies that target the extracellular domain of EGFR, such as cetuximab, and small molecule TKIs that target the catalytic domain of EGFR, such as gefitinib and erlotinib. TKIs are synthetic, mainly quinazoline-derived, low-weight molecules that interact with the intracellular tyrosine kinase domain of several receptors, including EGFR, and inhibit ligand-induced receptor phosphorylation by competing for the intracellular ATP-binding site (Ciardiello, 2000). TKIs show initial response rates of over 75% in mutant *EGFR*-NSCLC patients (Perez-Ramirez et al., 2015). In the Iressa Pan-Asia Study (IPASS), *EGFR* mutations were reported to be the strongest predictive biomarker for progression-free survival and objective response rate to first-line gefitinib versus carboplatin/paclitaxel (Fukuoka et al., 2011). However, after an initial marked response to EGFR TKIs, almost all patients inevitably relapse via development of acquired resistance within a median of 9–12 months (Riely and Yu, 2015; Santarpia et al., 2015). Because it was observed that TKIs induced drug resistance in *EGFR* mutant lung cancer, this indicates that genetic variation and tumor heterogeneity can act as intrinsic drivers for the acquisition of additional mutations upon drug treatment.

**Figure 2:**
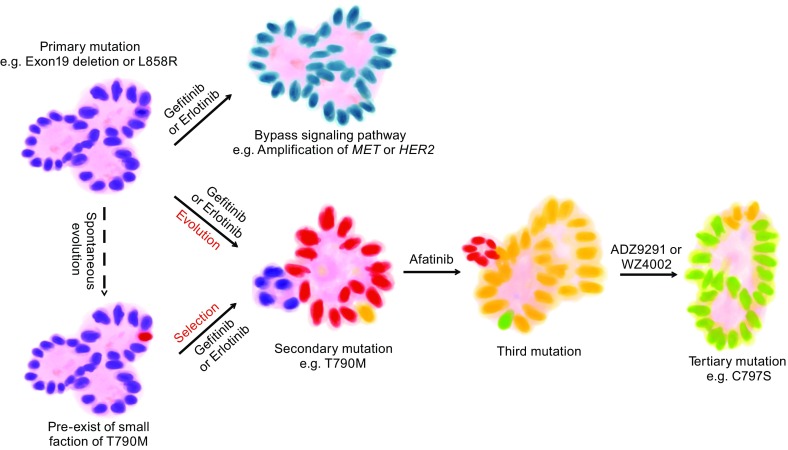
**Drug resistance in** ***EGFR*** **mutant lung cancer**. Lung ADC with *EGFR* mutation, such as exon 19 deletion or L858R substitution, can develop drug resistance after or even before drug treatment. In the first case, after being exposed to EGFR TKI, such as gefitinib or erlotinib, the tumor becomes resistant through bypass signaling pathway, such as amplification of *MET* or *HER2*. Also, under the pressure of inhibitor, the tumor can develop secondary mutation because of its genetic instability. As for the second case, the tumor has the capacity of spontaneous evolution before drug treatment. As a result, the tumor also develops secondary mutation. After drug treatment, the secondary mutation is subjected to selection and the cells with secondary mutation become a majority of the tumor. Admittedly, it is hard to diagnose in each tumor whether the secondary mutation has already existed before drug treatment or not. However in both cases, the tumor subsequently treated with the drug, which is targeted to the secondary or third mutation, finally develops drug resistance, manifesting as holding the third or tertiary mutation

One of the greatest therapeutic challenges is gatekeeper mutation that change the accessibility of a hydrophobic pocket critical for the binding of small molecule TKIs (Zhang et al., 2009). For example, the secondary mutation T790M increases the affinity of EGFR for ATP and renders the EGFR kinase inhibitors gefitinib and erlotinib less effective in displacing this ATP (Yun et al., 2008). The T790M mutation is responsible for half of EGFR TKI resistance (Wang et al., 2015). Strategies were developed to overcome this resistance such as the generation of the quinazoline irreversible EGFR TKI afatinib (Pao et al., 2005); however, high concentration of this inhibitor is required to inhibit the T790M mutation and acquired resistance to afatinib was also observed in clinical studies (Hashida et al., 2015). Although intensive preclinical studies show impressive results for third-generation mutant-specific EGFR inhibitors such as AZD9291, rociletinib, and WZ4002, drug resistance has still been observed. New tertiary mutations were identified such as C797S in AZD9291-resistant T790M-positive tumors and L718Q, L844V, and C797S in WZ4002-resistant tumors (Thress et al., 2015; Yu et al., 2015). Sequentially detected mutations in NSCLC samples depict the genetic alterations in response to the modified inhibitors. A recent study revealed that the acquired resistance caused by T790M could occur either by selection of pre-existing T790M-positive clones or via genetic evolution from initially T790M-negative drug-tolerant cells (Hata et al., 2016). Although heterogeneity-based tumor cell selection stands as a new mechanism of drug resistance, it determines cell identity through genetic alterations in early stage (Fig. 2).

Moreover, alternative signaling pathways, or so-called bypass tracks, can be activated in resistant cells bypassing the need for signaling from the target. An increasing recognized drug resistance mechanism occurs through a bypass tracks module. For example, 15% to 20% of EGFR TKI–resistant cases are mediated by amplification of the *c-MET* receptor tyrosine kinase. Activation of signaling downstream of *c-MET* is independent of EGFR and allows resistant cells to grow despite EGFR inhibition (Bean et al., 2007; Engelman et al., 2007). To prevent *MET* amplification, combination therapies of EGFR TKI and MET inhibitors have been applied in clinical trials. However, cancer relapse inevitably occurred. It was discovered that ATP-binding cassette subfamily B member 1 (ABCB1) overexpression, which was associated with cancer stem cell (CSC) properties and EMT, was involved in the acquired resistance to MET inhibitors. ABCB1 inhibitors could be a potential drug to fight against this resistance (Sugano et al., 2015) although this remains to be investigated (Fig. 2).

Furthermore, systematic genetic and histological analyses have been performed on tumor biopsies from 37 patients with drug-resistant lung adenocarcinoma carrying *EGFR* mutations (Sequist et al., 2011). Other than *MET* amplification, some resistant cancers showed unexpected genetic alterations including mutations in *PIK3CA* and amplification of *HER2*. Aberrant activation of PI3K/AKT signaling in the presence of EGFR kinase inhibitor can result in drug resistance (Engelman and Janne, 2008). Takezawa et al found that *HER2* was amplified in 12% of tumors with acquired resistance and 1% of untreated lung adenocarcinomas (Takezawa et al., 2012) (Fig. 2).

### Drug resistance of lung cancer with EML4-ALK fusion

**Figure 3:**
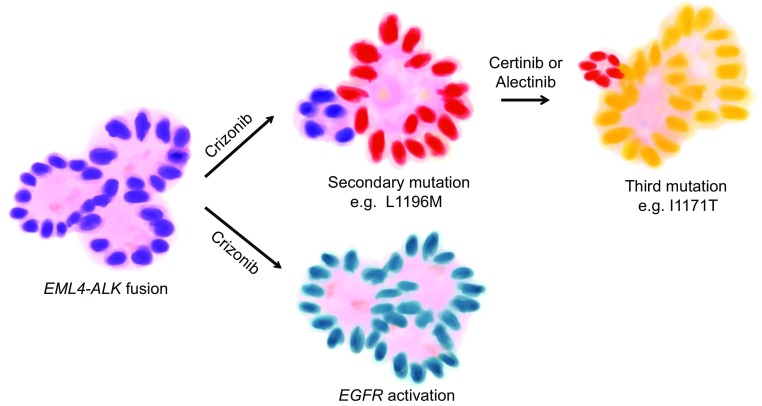
**EML4-ALK fusion and TKI resistance**. Similar to EGFR TKI resistance, lung ADC with *EML4-ALK* fusion gene also becomes resistant to ALK TKI in two major ways. One way is due to secondary mutation after crizonib treatment; and cells with third mutation dominate the tumor after being treated with second-generation ALK inhibitor. Another way is through EGFR activation in order to compensate the inhibitory effect of ALK inhibitor

Another example of drug resistance in lung cancer is anaplastic lymphoma kinase (ALK) target treatment. Echinoderm microtubule-associated protein-like 4 (EML4) and ALK (Choi et al. 2010) fusion protein is an oncogenic driver occurring in approximately 5% of NSCLC and leading to the expression of constitutively active ALK tyrosine kinase (Soda et al., 2007). Aberrant ALK activity activates mitogen-activated protein kinase kinase/extracellular signal-regulated protein kinase (MEK/ERK) and PI3K signaling pathways and promotes cell survival and proliferation (Shaw et al., 2013). Crizotinib is an ALK ATP-competitive inhibitor used as first-line therapy in the treatment of advanced NSCLC harboring *ALK* rearrangement. Unfortunately, responses to this agent are not long-lasting. Genetic alterations and activation of bypass tracks are two main mechanisms of acquiring resistance. Secondary mutations in *ALK* TK domain as well as *ALK* fusion gene amplification have been identified (Doebele et al., 2012; Katayama et al., 2012). L1196M, a gatekeeper mutation, has been detected, as well as L1152R, C1156Y, I1171T/N/S, F1174L/C/V, V1180L, G1202R, S1206Y, and G1269A mutations (Iams and Lovly, 2015). In crizotinib-resistant tumors, several distinct bypass tracks mediating resistance have been reported. Among them, activation of EGFR has been identified in several independent studies. As revealed by studies from the Massachusetts General Hospital, 5 out of 9 resistant patient samples have EGFR activation compared to sensitive samples. This finding has been further confirmed by *in vitro* experiments (Sasaki et al., 2011; Katayama et al., 2012; Yamaguchi et al., 2014). Katayama et al also found high-level of *c-KIT* gene amplification in 2 out of 18 resistant samples. Further studies showed that c-KIT overexpression required stem-cell factor to promote resistance and that resistance could be overcome by combined imatinib and crizotinib treatment (Katayama et al., 2012). Upregulation of insulin like growth factor receptor 1R (IGF-1R) and SRC activity have also been detected in ALK TKI resistance (Crystal et al., 2014; Lovly et al., 2014). Furthermore, P2Y receptors were reported to mediate ALK inhibitor resistance and protein kinase C played an important role in this process (Wilson et al., 2015) (Fig. 3).

Based on the exploration of the resistance mechanisms, multiple second-generation ALK inhibitors functioning as first-line therapies and crizotinib resistance therapies are currently applied in clinic, such as ceritinib, or are in clinical trials, such as alectinib, brigatinib, X-396, PF-06463922, and TSR-011 (Iams and Lovly, 2015; Wilson et al., 2015). However, resistance to second-generation ALK inhibitors has also been observed. Katayama et al found a novel V1180L gatekeeper mutation from a cell line model and a novel I1171T secondary mutation from a patient who developed resistance to alectinib (Katayama et al., 2014). Another study suggested that inhibitors of heat shock protein 90 (Hsp90), such as 17-DMAG, ganetespib, and IPI-504, may overcome ligand-triggered resistance to second-generation ALK inhibitors (Tanimoto et al., 2014). However, resistance to Hsp90 inhibition has also been discovered. Expression of P-glycoprotein has been reported to be associated with resistance to 17-DMAG (Kim et al., 2015) (Fig. 3).

### Genetic evolution and heterogeneity

It is generally accepted that tumors are monoclonal in origin. That is, only a single cell can cross over the border from normalcy to malignancy to become the ancestor of the cells in a tumor mass. As a result, all cells in a tumor are expected to share common genetic or biochemical markers (Weinberg, 2013). However, it is also known that tumors are composed of several subpopulations with genotypic and phenotypic heterogeneity. At initiation, the tumor is a relatively homogeneous collection of cells that soon become heterogeneous due to the continuous acquisition of new mutations. This acquisition is mostly due to the development of genetic instability. The clonal evolution model suggests that the genetic evolution among cancer cells is responsible for cancer progression and heterogeneity in phenotype, function, and response to therapy (Hockel and Vaupel, 2001).

Tumor initiation and progression are dependent on rare, stochastic mutations that create genetic variability in a cell population. Most of the spontaneous mutations are neutral or deleterious, while disadvantage mutations will be discarded, and neutral mutations can still retain. Mutations that confer competitive advantages, such as increased proliferation and survival in the restrictive microenvironment, can be selected for by Darwinian forces. As a consequence, these cells have an advantage in outgrowth and eventual dominance in the local tissue environment. Normally, a majority of the tumor cells undergo several successive mutations and Darwinian selection after the initiating mutation. This process is called clonal succession, on which the clonal evolution model is based (Weinberg, 2013). However, a minority of tumor cells may harbor only one or two advantageous or neutral mutations. This provides genetic heterogeneity within a tumor. Additionally, cells within different parts of the tumor could experience different selective pressures before drug treatment, such as differential interactions with the extracellular matrix, gradients of oxygen, nutrition, growth factors, and blood supply. This differential exposure could be another cause of tumor heterogeneity (Marusyk and Polyak, 2010).

Following drug treatment, the primary stress afflicting tumor cells is caused by the drug and genetic heterogeneity might be a reasonable explanation of drug resistance. Although it is accepted that resistant properties occur after pharmacological treatment, researchers have found that some resistance-related mutations exist prior to treatment (Denis et al., 2015; Gurden et al., 2015). There is also the possibility that previously advantageous mutations may no longer benefit the tumor proliferation or survival, but that previously neutral mutations harbored by a very small fraction of the tumor cells become beneficial under drug exposure. Drug treatment becomes the catalyst for the accumulation of certain clones that then become detectable. The preexistence of EGFR TKI resistant cells have been validated in cell lines as well as in primary human lung tumors through high-throughput FISH analyses and peptide nucleic acid-clamping PCR or ultra-sensitive droplet digital PCR (Turke et al., 2010; Oh et al., 2011; Watanabe et al., 2015). Darwinian selection will allow tumor cell survival in patients with pre-existing resistant cells, even if it is a minimal subgroup, when drugs are administered (Gerlinger et al., 2012; Turtoi et al., 2015). On the other hand, if there are no pre-existing advantageous mutations against the drug treatment, the tumor will undergo regression. However, during this period of drug treatment, residual cells may still mutate. Tumor cells that adapt to drug treatment by acquiring additional mutations will dominate the tumor cell population rapidly. Both pre-existing and later evolving mutations could lead to detectable genetic alterations. Changes in the cell population embody the characteristic of cancer plasticity, which could explain how genetic alterations contribute to EGFR or ALK resistance.

## Modulating plasticity and heterogeneity: an opportunity for cancer treatment

**Figure 4:**
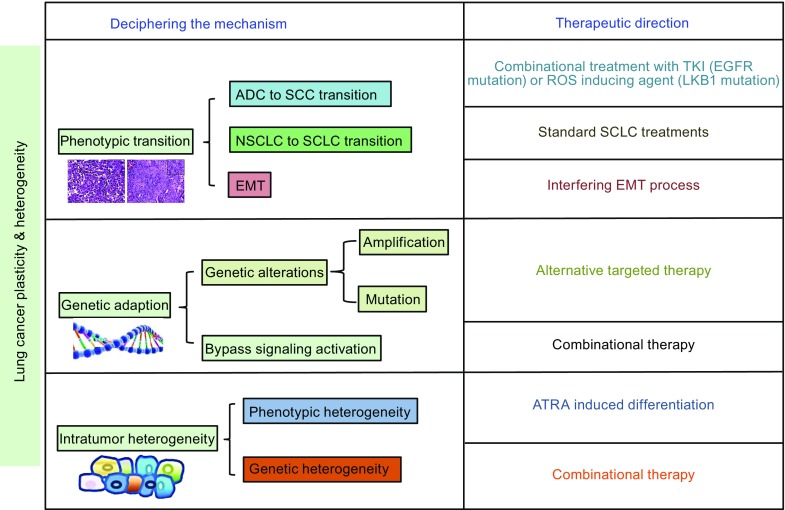
**Summary of mechanisms of lung cancer plasticity, heterogeneity and the correlated therapeutics**. This model illustrates the guidance of lung cancer plasticity and heterogeneity in clinical use, especially in drug resistance upon target therapies. Different therapeutic strategies depend on the unique mechanisms. Histologically, transition from ADC to SCC and switch from NSCLC to SCLC have been found broadly. Complete transition leads to the using of transited tumor therapy while combinational treatment is applied to partial transformation. EMT is a special aspect of phenotype alteration. It shows morphological plasticity of lung cancer with the switch of biological markers. Interfering EMT process can be a bright way to inhibit certain kind of drug resistance. In addition to histological transition, genetic adaption provides an evident proof for lung cancer heterogeneity. As the intensive study of cell and molecular mechanism, gene alteration and abnormal activation have been revealed in the drug-surviving cancer cells. More target-pointed drugs are needed, substitutionally or combinationally. Intratumor heterogeneity reinforces the composition of lung cancer that changes for adapting to the environment. A typical illustration is the acquirement of stem cell-like property, against which ATRA is an effective therapy. In terms of targeting genetic heterogeneity, identification of advantageous clones and application of combinational therapy could provide a better outcome for lung cancer patients

Cell intrinsic and cell extrinsic features contribute to tumor heterogeneity, which aids cancer cells in acquiring functional capabilities that allow the malignant cells to survive, proliferate, and disseminate through genetic alterations, epigenetic modifications or subtype transdifferentiation. Cell extrinsic properties arise from factors in the microenvironment including the composition of the extracellular matrix and factors sequestered to its constituents, the tumor’s ability to recruit adequate blood supply, and the recruitment of stromal cells that aid in tumor growth (Marjanovic et al., 2013). These diverse aspects help cancer cells fight drug treatment and eventually develop resistance. However, studying tumor plasticity and heterogeneity provides us with more opportunities to fully understand the processes and molecular mechanisms that underlie drug resistance. It is therefore reasonable to be optimistic about the future of lung cancer treatment benefiting from an increased knowledge of plasticity and heterogeneity (Fig. 4).

Opportunities brought by cancer plasticity can be manifested in treatment. Loss of stem properties in tumor initiating cells (TICs) (Lovly et al.) can be beneficial for drug treatment with fewer concerns regarding clinical relapse. Cancer stem cell differentiation is a new hot spot for such studies. All-trans retinoic acid (Miller et al. 2004) is an agent that can induce differentiation and may be effective against residual tumor cells that are resistant to drug treatment. Moro et al found that several cycles of all-trans retinoic acid (ATRA) and cisplatin treatment are able to stably reduce lung TIC and dissemination (Moro et al., 2015). The histone deacetylase inhibitors senistein and C-phycocyanin have been reported to strengthen the anti-cancer affects of ATRA and reduce the metastatic potential of lung cancer (Zhou et al., 2012; Cheng et al., 2015; Greve et al., 2015; Li et al., 2015a). ATRA also works in the development of drug-resistant neuroblastoma both *in vitro* and *in vivo* (Matthay et al., 1999; Merrill et al., 2004). In advanced hepatocellular carcinoma, ATRA also helps against resistance to sorafenib, which is considered as the first-line treatment of the malignant disease (Guan et al., 2015). These studies suggest that depriving stem cell function might be universal and effective in cancer treatments, including in drug resistance in lung cancer (Fig. 4).

Moreover, the inhibition or interference of the transdifferentiation process may strengthen the effect of TKI treatment *EGFR* mutant lung ADC. In a *Lkb1*-deficient lung cancer mouse model, the quenching of ROS has been validated to functionally inhibit the ADC-to-SCC transdifferentiation (Li et al., 2015b). Although validation of phenotypic transition in human NSCLC is challenging, the potential for the combination treatment of ROS scavengers with transition inhibitors to overcome drug resistance is high. Additionally, impairing the ability of tumor cells to eliminate accumulated oxidative stress during metabolically controlled phenotypic transitions may sensitize tumors to ROS inducing reagent. Awareness of lung cancer plasticity may affect treatment regiments to maximize pathological response. If transdifferentiation can be detected early, corresponding changes to therapy may benefit patients. On the other hand, a forced transdifferentiation to a complete transformation may make tumors more sensitive to chemotherapy and radiation than the original tumor. In this manner, patients may achieve longer period of disease control and survival via effective chemotherapy and radiotherapy (Fig. 4).

Plasticity may allow cancer cells to revert back to normal cells. The discovery of the reversal phenomenon dates back to the 1970s. It was observed that the injection of blastocysts into malignant mouse embryonal carcinoma cells reverted them back into normalcy (Mintz and Illmensee, 1975). This shows the impact of the microenvironment on cancer progression. Cancer cell reversal to non-malignant cells has been observed in various types of cancers through transplantation into a normal adult or embryonic tissue (Lee et al., 2005; Krause et al., 2010; Bischof et al., 2013; Brock et al., 2015). This highlights a potential new approach to cancer treatment helping cancer cells transit back into the normal state. Recently, the development of systems biology has allowed for a more efficient exploration of phenotype switching and identification of essential molecular targets (Brock et al., 2015). While there are no current applications of cancer-normalcy-transition treatment, new mechanisms are continuously being uncovered. The study of normalcy reversal could lead to the development of novel cancer therapies one day.

Increasing evidence has shown that inhibition of EMT may be a possible mechanism of drug resistance. Suppression of the master EMT-inducer zinc finger E-box-binding homeobox 1 (ZEB1) significantly enhanced the chemosensitivity of docetaxel-resistant ADC *in vitro* and *in vivo,* and ectopic expression of ZEB1 increased chemoresistance (Ren et al., 2013). Furthermore, through the screening of established small anti-cancer molecules in the *EGFR* mutant NSCLC HCC827 cell line and a corresponding mesenchymal derivative cell line, Wilson et al. found that dasatinib, an Abl/Src inhibitor, was a potential candidate for treatment of erlotinib resistance associated with EMT (Wilson et al., 2014). A forced EMT experiment also helps finding of agents with epithelial CSC-specific toxicity. The trial is based on the study of epithelial CSCs which are enriched in EMT process. The candidate agent Salinomycin can lead to the loss of expression of breast cancer stem cell genes in patients (Gupta et al., 2009). However, recent research shows that non-cancer stem cells have the potential to dedifferentiate into cancer stem cells (Chaffer et al., 2011). A major limitation to the use of agents with CSC-specific toxicity is the possibility for the non-stem cells to convert into cancer stem cells. However, further research may identify new strategies to overcome these conflicts, such as finding new agents targeting both cancer stem cells and non-stem cells, or drugs specifically point to non-cancer stem cells and cancer stem cells respectively (Gupta et al., 2009).

Heterogeneity, particularly intratumoral heterogeneity, increases the challenges of cancer subtype classification. It may lead to the biased analysis of single tumor-biopsy samples since the sample may represent only a part of the tumor genomic landscape (Gerlinger et al., 2012). This represents a potential obstacle to successful biomarker discovery, validation, and application (Dibben et al., 2012; Gerlinger et al., 2012). Heterogeneity may also partly contribute to the failure of personalized medicine. This highlights the need for biomarker studies, macro-dissections, and increased number of biopsies per patient (Dibben et al., 2012). Although no consensus has been reached, optimized biopsies may lead to better-targeted therapies or combined treatments (Fig. 4).

## Conclusion

In the clinical treatment of lung cancer, drug resistance remains a frustrating issue. In this review, we attribute this problem to lung caner plasticity and genetic heterogeneity at both the histological and genetic levels. More specifically, these mechanisms include secondary mutations, bypass signaling pathway, histological transformation from NSCLC to SCLC, transdifferentiation from ADC to SCC, cancer stem cell differentiation, and the EMT program. Cancer cell plasticity and genetic alterations further contribute to the tumor heterogeneity of lung cancer, serving as selective material under restrictive conditions, such as drug exposure, tumor microenvironmental and metabolic stress. In these situations, only more adaptive cells can survive and accumulate in a tumor. As a result, drug resistance eventually develops. However, we can hope to be inspired by understanding and exploiting the mechanisms of plasticity. For instance, we can utilize oxidative stress scavengers to inhibit transdifferentiation, apply oxidative stress-inducing agents to ROS sensitive tumors, and use certain agents to limit the process of EMT and cancer stem cell differentiation. Moreover, plasticity-induced heterogeneity emphasizes the importance of the collection of more representative biopsies and the discovery of more optimized biomarkers for pathological diagnosis. Lung cancer plasticity and heterogeneity remains a significant challenge, but there is a real potential to exploit it in the fight against drug resistance.
